# “Like you are fooling yourself”: how the “Stoptober” temporary abstinence campaign supports Dutch smokers attempting to quit

**DOI:** 10.1186/s12889-019-6833-y

**Published:** 2019-05-07

**Authors:** Sigrid A. Troelstra, Anton E. Kunst, Janneke Harting

**Affiliations:** Department of Public Health, Amsterdam Public Health Research Institute, Amsterdam UMC, University of Amsterdam, Meibergdreef 9, 1105 AZ Amsterdam, the Netherlands

**Keywords:** Smoking cessation, Intervention, Stoptober, Qualitative research, Temporary abstinence campaigns

## Abstract

**Background:**

The Stoptober temporary abstinence campaign challenges smokers to engage in a collective quit attempt for 28 days. The campaign is based on social contagion theory, SMART (i.e., Specific, Measurable, Attainable, Realistic and Time-sensitive) goal setting and PRIME (i.e., Plans, Responses, Impulses, Motives and Evaluations) theory. Although Stoptober was found to yield impressive 28-day quit rates, relapse rates remained substantial. Therefore, we examined how Stoptober supported smokers in their attempt to quit and how the campaign’s effectiveness could be strengthened.

**Methods:**

In 2016, we conducted semi-structured interviews with 23 Stoptober participants in the Netherlands. Data were analyzed thematically.

**Results:**

Respondents explained how social contagion-based components had familiarized them with Stoptober, motivated them to participate, and created a pro-smoking cessation social norm. Setting SMART goals was reported as “fooling yourself”, since it distracted respondents from their goal of quitting for good and helped them perceive that temporary abstinence was achievable. Respondents also illustrated the usefulness of PRIME theory. They typically used an individual selection of available supports that varied over time. To achieve long-term abstinence, respondents expressed the need for additional social network support and interactive, personalized and professional support during and after the campaign.

**Conclusions:**

Stoptober supports smokers in their attempts to quit and generally according to the campaign’s theoretical principles. Added to available evidence, this finding supports the continuation and wider implementation of Stoptober, while connecting the campaign to social networks and regular smoking-cessation services to help improve long-term abstinence rates.

**Electronic supplementary material:**

The online version of this article (10.1186/s12889-019-6833-y) contains supplementary material, which is available to authorized users.

## Background

Tobacco use is a main cause of death worldwide [1]. Although the prevalence of smoking in Western European countries is decreasing [2], the increase in the percentage of non-smokers has slowed and is mainly limited to higher socioeconomic strata [3]. This development indicates that there is still ample room for cessation programs that are effective and able to reach a substantial number of smokers [4].

The mass media smoking-cessation campaign Stoptober is a promising example of such a program [5]. Stoptober is a temporary abstinence campaign that challenges smokers to quit smoking for 28 days in October. A population-level evaluation of the 2012 edition of the campaign in England revealed a 50% increase in the national attempt-to-quit rate [5]. In October 2012, this attempt-to-quit rate was 9.6%, compared to 6.6% for the rest of 2012, and 6.4% in Octobers of the previous years [5]. Based on an observed effect of 350,000 additional quit attempts in England, it was estimated that Stoptober could lead to substantial behavior change and public health impact [5]. Stoptober’s potential was supported by a prospective cohort study of participants in the 2016 edition of the Dutch version of the campaign [6]. This study estimated quit rates of 40–60% (two months post-campaign) among the 53,000 program participants. The impressive quit rates and the still substantial relapse rates estimated by these evaluation studies, raise the questions of how exactly the Stoptober campaign achieved success, and how the program can be improved.

Theoretically, the success of the Stoptober campaign may be attributed to the use of three key social-psychological principles [5]. The first principle is social contagion theory [7]. Based on this theory, Stoptober makes use of social networks to disseminate and intensify the campaign’s message. This social network approach aims to create a collective quit attempt, as subgroups of interconnected smokers may prefer to stop smoking together [5]. By making use of both traditional and new mass media channels, and focusing on positive messaging (e.g., “Stop smoking for 28 days and you’re five times more likely to quit for good”), Stoptober aims to create a social movement and normalize smoking cessation [5].

The second principle that Stoptober applies is SMART (i.e., Specific, Measurable, Attainable, Realistic and Time-sensitive) [8]. Difficult changes, such as stopping smoking, are expected to be more easily achieved if the behavioral goals are SMART-formulated. In Stoptober, this is illustrated by challenging smokers to stop smoking, which is behavior-specific and measurable, for a period of 28 days in October. This can be viewed as an attainable, realistic and time-sensitive intermediary goal towards becoming a permanent non-smoker.

The third key principle for Stoptober is PRIME (i.e., Plans, Responses, Impulses, Motives and Evaluations) theory [9]. PRIME theory is a comprehensive theory of motivation that assumes: (1) behavior is determined from moment to moment by a wide variety of motivational inputs; (2) a motivational system is unstable; (3) and therefore, maintaining certain behavior requires a constant balance of inputs. Programs that aim to achieve behavior change (e.g., aim to stimulate smokers to quit), should offer a range of support that triggers the whole motivational system rather than only elements [9]. This support should both weaken the motivational powers that cause the behavior (e.g., smoking) and create new sources of desire and control to refrain from that behavior (e.g., quitting smoking). Based on the PRIME principle, Stoptober offers an elaborate support package (examples of program components are presented in Table 1).

**Table 1:** Key psychological principles underpinning Stoptober’s program components

Psychological principle^a^	Program components	Theoretical methods^b, c^
Social contagion Social networks can facilitate the spread of attitudes and behavior and increase the reach and intensity of a message. Stoptober used traditional and new mass media channels to create a mass quitting trigger and actively support a social movement around stopping smoking. Positive messaging was used to build engagement, enhance message dissemination and normalize quitting behavior. Stoptober aimed to increase support for the campaign, and to motivate smokers to participate and encourage others to do so too, since interconnected groups of smokers often try to quit together.	Press exposure, television and radio broadcasts include the message that Stoptober is coming up and all smokers should participate and try to quit smoking temporarily on the same date. Non-smokers are encouraged to support smokers in their participation.	Increase awareness of Stoptober Increase awareness of the advantages of smoking cessation Persuasive communication Mobilizing social network support Increasing feelings of self-efficacy
	The Stoptober camper travels to various parts of the country to reach a large number of people. The camper personnel provide information about smoking cessation and encourage participants to take part in Stoptober.	Increase awareness of the advantages of smoking cessation Increased awareness of Stoptober Persuasive communication Increasing feelings of self-efficacy
SMART goals SMART (Specific, Measurable, Attainable, Realistic and Time-sensitive) goals help people to achieve a difficult behavior changes, such as stopping smoking for good. Therefore, Stoptober challenged smokers to set an intermediary goal, i.e., to stop smoking for a time-limited period. This goal may be relatively easy to achieve, and once achieved, it substantially increases the chances of becoming a permanent non-smoker.	Set time and duration of collective cessation attempt. Stoptober challenges smokers not to smoke for 28 days during the month October.	Goal-setting theory Implementation intentions Increasing feelings of self-efficacy
PRIME theory PRIME theory is a comprehensive theory of motivation. Behavior is determined from moment to moment by a wide variety of motivational inputs, while the motivational system is unstable and requires constant balancing of inputs to maintain a certain behavior (e.g. smoking). Programs that aim to achieve behavior change, e.g. quitting smoking, should offer a range of support that triggers the whole motivational system rather than single elements.^d^ This support should both weaken the motivational powers that cause the behavior (e.g. smoking) and create new sources of desire and control to refrain from that behavior (e.g. quitting smoking). Therefore, Stoptober offered an elaborate support package, consisting of Twitter messages, ambassadors, video diaries, a Facebook community, social media profile logos and an app. This support package aims to decrease the motivation to smoke and create new desires to quit smoking.	Subscription on the website to have access to the Stoptober app, a free magazine and newsletters	Goal setting theory Implementation intentions
	Email messages with tips, progress, motivational messages and news	Positive reinforcement Persuasive communication Increasing feelings of self-efficacy
	Bracelets to wear during the campaign to remind participants not to smoke and demonstrate their commitment to others	Public commitment Social support Positive reinforcement
	Twitter account wherein Stoptober frequently posts positive and encouraging messages for the participants	Positive reinforcement Persuasive communication Belief selection
	Well-known ambassadors participate in Stoptober, try to gain publicity for the program through their media presence and serve as examples for other participants.	Increase awareness of Stoptober Persuasive communication Mass media role modeling Provide opportunities for social comparison
	Video diaries of Stoptober participants on YouTube and Facebook.	Social support Role modeling Opportunities for social comparison
	Facebook page wherein Stoptober frequently posts positive and encouraging messages for the participants and where they can share their accomplishments and struggles. Participants can share tips, compliments and encouragement.	Positive reinforcement Persuasive communication Belief selection Mobilizing social networks Opportunities for social comparison Advice on relapse prevention: • Counterconditioning • Cue altering • Stimulus control • Planning coping responses • Resisting social pressure • Providing contingent rewards Increasing feelings of self-efficacy
	Stoptober logos for Facebook profile pictures to notify friends and family of their participation.	Public commitment Mobilizing social support Mobilizing social networks
	Stoptober app keeps track of abstinence, amount of money saved and number of unsmoked cigarettes. Participants can earn achievement badges and press an ‘emergency’ button to help with cravings.	Self-monitoring of behavior Positive reinforcement Persuasive communication Advice on relapse prevention: • Counterconditioning • Cue altering • Stimulus control • Planning coping responses • Resisting social pressure • Providing contingent rewards Increasing feelings of self-efficacy

^a^Brown J, Kotz D, Michie S, Stapleton J, Walmsley M, West R. How effective and cost-effective was the national mass media smoking cessation campaign ‘Stoptober’? Drug Alcohol Depend. 2014;135(100):52–8

^b^Eldredge LKB, Markham CM, Ruiter RAC, Fernández ME, Kok G, Parcel GS. Planning Health Promotion Programs: An Intervention Mapping Approach. 4 ed.: Wiley; 2016

^c^Michie S, Richardson M, Johnston M, Abraham C, Francis J, Hardeman W, et al. The Behavior Change Technique Taxonomy (v1) of 93 Hierarchically Clustered Techniques: Building an International Consensus for the Reporting of Behavior Change Interventions. Annals of Behavioral Medicine. 2013;46(1):81–95

^d^West R, Brown J. Theory of Addiction: Wiley; 2013

The aim of our qualitative inventory of Stoptober participant experiences was to understand how the campaign supported smokers in their attempts to quit and how they thought the program could be improved to prevent smoking relapses during and after the campaign. We intended to further strengthen Stoptober’s evidence-base and contribute to the campaign’s effectiveness and wider implementation.

## Methods

### Design

We conducted a qualitative study shortly after the 2016 Stoptober program in the Netherlands ended. We retrospectively interviewed a sample of participants to understand the campaign’s working mechanisms [10], including how the program’s components had or had not brought about the intended effects based on the three key theoretical principles. We held semi-structured interviews and performed a thematic analysis [11]. We followed the criteria for reporting qualitative studies (COREQ).

### Participant selection

More than 53,000 individuals subscribed to the 2016 Dutch Stoptober. Respondents for our study were recruited by convenience sampling through both the program’s Facebook page and email at the end of October and the beginning of November to avoid interference with the simultaneous effect study [6]. Participants were asked to contact the first author [ST] if they were willing to take part in an interview about their experiences during campaign and if so, they would receive a gift voucher of 20 euro. Participants were approached in an open, non-normative way, especially with regard to their success or failure in the campaign. In general, 20 to 25 interviews are sufficient to reach information saturation [12]. Therefore, we stopped recruitment after 24 interviews were scheduled. Most respondents had approached us via the Facebook solicitation. Since one interview was cancelled due to logistics, the final sample included 23 Stoptober participants.

### Setting

The first author [ST], a female doctoral student with a Master of Science degree trained in qualitative research methods, conducted the interviews in November (*n* = 17) and December 2017 (*n* = 6), at a location of the respondent’s choice: participant’s home (*n* = 18) or workplace (*n* = 3), researcher’s office (*n* = 1), or a café (*n* = 1). Before the start of each interview, the interviewer tried to establish rapport by making small talk and having a coffee. The interviewer did not have a prior relationship with the participants. No third persons were present during the interviews. Most respondents were female, 30–60 years old, and first-time participants in Stoptober (Table 2). Before the campaign, respondents typically smoked about a one pack of cigarettes a day. Two respondents relapsed during Stoptober and two others relapsed after Stoptober. About half of the sample had used additional support to stop smoking (e.g. varenicline, nicotine replacement therapy, e-cigarettes).

**Table 2:** Characteristics of study population

Respondent	Abstinent during Stoptober	Quit smoking at time of interview	Former daily cigarette consumption	Former participation	Additional support
1	Yes	No (reduction)	1 pack	Yes	–
2	No (reduction)	No (reduction)	1f.5 pack	No	–
3	Yes	Yes	1 pack	No	Varenicline (prescribed by GP)
4	Yes	Yes	e-cigarettes	No	e-cigarettes (self-obtained)
5	Yes	Yes	1 pack	No	e-cigarettes (self-obtained)
6	No	No	1 pack	No	e-cigarettes (self-obtained)
7	Yes	Yes	1.5 pack	No	–
8	Yes	Yes	1 pack	No	Hypnosis (self-organized)
9	No (reduction)	Incidental smoking	1 pack	No	Varenicline (prescribed by GP)
10	Yes	Yes	More than 1 pack	No	–
11	Yes	Yes	10 cigarettes	No	Varenicline (prescribed by GP)
12	Yes	Yes	1 pack	No	–
13	Yes	Yes	1 pack	No	–
14	Yes	Relapsed, a few puffs	1 pack	Yes	NRT^a^ (self-obtained)
15	Yes	Yes	About 1 pack	No	
16	Yes	Yes	25 cigarettes	Yes	Self-help book (self-obtained)
17	Yes	Yes	1 pack	Yes	–
18	Yes	Yes	1 pack	No	NRT^a^ (self-obtained)
19	Yes	Yes	1 pack	No	–
20	Yes	Yes	1 pack	Yes	Support group (organized with colleagues)
21	Yes	Yes	More than 1 pack	No	–
22	Yes	Yes	10–15 cigarettes	No	Individual counselling with smoking cessation psychologist (approached by psychologist)
23	Yes	Yes	1 pack	No	–

^a^NRT: nicotine replacement therapy

### Data collection

Semi-structured interviews were chosen to allow participants to express their experiences and ensure the main research themes were addressed [13]. The interview guide included topics such as current smoking status, Stoptober’s role in the attempt to quit, experiences with specific program components, changes in psychosocial determinants of smoking (i.e., attitude, social norms, social support, self-efficacy, habit and identity), expectations regarding future smoking status, and ideas for improving Stoptober. An English version of the interview guide is provided in Additional file 1. Interviews lasted between 20 and 60 min (average 45 min). The interviews were audiotaped after receiving consent from the respondents and were transcribed verbatim.

### Data analysis

The interviews were thematically analyzed [11] in MAXQDA [14]. First, ST and JH individually coded two “rich” interviews. They used the interview guide as a foundation for the analysis, but open coding was also allowed. Based on their findings, codes were added for program components and coping strategies. Second, ST systematically coded all other interviews with the final coding scheme (Table 3). All coding by ST was checked by JH. Third, ST summarized the main findings per theme. Finally, ST and JH categorized these findings into the three key theoretical principles of Stoptober (i.e., social contagion theory, SMART goals, and PRIME theory). Additional file 2 reflects the categorization part of the data analysis. Disagreements were discussed until consensus was reached.

**Table 3:** Coding scheme

Prior to campaign	Rationale to quit smoking	
	Rationale for participation in Stoptober	
	Smoking behavior	
Experiences during campaign	Difficult moments	Withdrawal
		Addiction
		Stress
	Positive experiences	
	Additional support	Medication
		Other support
Strategies to break habit	Reduce smoking	
	Farewell ritual	
	Role modeling	
	Relapse prevention	
	Compliments	
	Rewards	
	Anticipated regret	
	Resistance to social pressure	
	Public commitment	
	Counterconditioning	
	Substitute behavior	
	Cue avoidance	
	Cue altering	
	Self-talk	
	Self-management	
	Goal setting	
Behavioral determinants	Attitude	
	Social influence	Social support
		Injunctive norm
		Descriptive norm
		Social pressure
	Self-efficacy	Confidence in success
	Habit	
	Identity	
	Motivational strength	
Future	Confidence in maintaining abstinence	
	High risk situations	Action plans
Intervention components	General	Other
	Facebook	
	App	
	Mass media	
	Ambassadors	
	Set date and time	
	Needs	

## Results

### Support experienced

The Stoptober message was spread throughout the Netherlands with high intensity through various traditional and new mass media channels. Our respondents typically explained how this *social contagion strategy* had familiarized them with the program and motivated them to participate.

*Before the campaign you frequently saw commercials on the television with the announcement that Stoptober was coming up. And [ … ] because I saw it so often, I thought: “This might be something, should I participate or not?” And eventually, after seeing it several times, I thought: “Let’s just try it, I will do it.” [R02]*

*It makes you think about it. [ … ] I saw that many people had registered. It would be a nice stimulus, a good moment. [ … ] So many smokers are going to quit, so I should be able to succeed again too. [R07]*

These accounts also indicate how the mass media approach may have contributed to the normalization of smoking cessation and strengthened the respondents’ social support and self-efficacy to quit.

Many respondents explained how the *SMART goal* set by Stoptober had assisted them in their current attempt to quit. For instance, they mentioned that appointing October as the month to stop smoking collectively had made it easier for them to set a quit date and the defined quit period of 28 days had made their attempt look more achievable.

*And the date. You often try to set a date. Most people do it [try to quit] at New Years. But we just didn’t have a date anymore because we’d tried so many times already. [ … ] With New Years, you don’t participate together with so many others. [R18]*

*Look, you can always give it a try. It’s a month, it is not like you are quitting entirely, because then you would dread it. You would feel stressed and think: CIGARETTE!!!. This is ideal. [R02]*

Although experienced as improving their self-efficacy at the start, respondents also stated that at some point in time, they had realized their temporary quit goal was only a trick to distract themselves from the ultimate goal of quitting for good.

*It’s just like you are fooling yourself, actually. It is quitting for 28 days, with the idea: “After 28 days I can smoke again”, and that is what makes you hold on. [ … ] [But] eventually you realize that quitting for 28 days, well, you don’t accomplish anything with that. [Because] it’s temporary. [R19]*

The respondents’ reports clearly illustrate how the variety of support offered by Stoptober (in line with *PRIME theory*) had supported them in their attempt to quit by allowing them to make an individual selection based on their personal needs and preferences. *“You use whatever suits your needs.”* [R12] For example, while some respondents thought the Stoptober app had been most supportive, others found that the Stoptober Facebook page had offered the best help.

*I have to say, that [the app] really works. You get notifications like “you saved this much [money]”, “you have received this badge” or “you can taste better now”. [ … ] And it was true and it made me think: “YES, let’s go for the next one. For me, that was a great tool”. [R15]*

*For me the stories on Facebook were of greater use. So, they [Stoptober] posted a message [on the Facebook page]. Many people reacted to this, and from that you could read: “Well, they are also going through a difficult time”. [ … ] And when some said: “I smoked a cigarette”, others would say [ … ]: “Don’t worry, never mind, just keep on going”. [ … ] It is really nice, because you can share [experiences]. [R14]*

These accounts indicate that the Stoptober app primarily served as a self-monitoring tool, while Facebook mainly provided a platform for exchanging experiences, making social comparisons, and providing and receiving social support.

Respondents frequently made use of additional pharmacological, behavioral or social support during the campaign (Table 2), however they still reported a need for further support from Stoptober during and after the campaign.

### Further need for support

The respondents reported that their needs changed over time and the support did not adapt accordingly. For instance, some respondents explained that the frequent posts from Stoptober that had been helpful at the start, gradually became counterproductive to their attempt to quit, since the posts reminded them too much of smoking.

*At a certain point, I felt that it [the Stoptober posts] worked negatively. [ … ] In the beginning you are always thinking of smoking. You don’t want that anymore. And when you think of smoking less and less, you receive messages on smoking cessation once in a while. So [ … ] after three weeks, I thought: “No, [..] not all those pop-ups and notifications.” I switched them all off. [R03]*

Another incongruity was reported when about halfway into the campaign, Stoptober started to communicate through the app that the participants had (almost) achieved cessation, while the respondents stated that for most of them, this was not yet the case.

*But, after that I realized that Stoptober was like: “Yes, you did it, already two weeks!” [ … ] I saw that people were quite angry about this. [ … ] The addiction is gone, but if I think of how strong that thing is what’s still in your head. That is real too. I think that maybe people, [ … ] maybe also Stoptober [ … ], underestimate how strong that part still is. [R17]*

The respondents reported a need for adjusted support, for instance by offering a broader variety of announcements, feedback messages and role-model stories, or including more interactive and personal elements.

*A bit more interaction. [ … ] That [Stoptober] will make itself heard a bit more, except for posting a message. Also replies on all responses. Will enter into debates. [ … ] That they will react with positive feedback or advice. [R08]*

*Yes [the emergency button of the app], did not help very much. But, I wouldn’t know how to change it. That’s really difficult right? I would prefer having someone [a real person] jump out and saying: “DON’T DO IT!” [laughter]. [R21]*

Respondents also referred to additional social network components. While some suggested connecting Stoptober to other online social networks (e.g. Instagram), more prominent were the respondents’ desires for additional offline social networks.

*Find people within Stoptober with whom you can talk before or after. [ … ] I think that this will also be a motivation for people to remain abstinent. [ … ] I would have liked it if there was an activity nearby for people who had also quit smoking. [ … ] In that way, you get to know someone and can support each other. [R05]*

*I participated last year too, then I did not succeed. [ … ] So, I went looking, beforehand. [ … ] How nice would it be to quit together with others? [ … ] A colleague of mine, [ … ] was also thinking about it [quitting]. I thought, well then, let’s just organize it collectively for all coworkers. That went really well. We have all quit smoking. [R20]*

This need for offline social networks was explained by the lack of social support and the strong pro-smoking social norm that respondents mostly experienced from their personal social networks.

*I’m far away from my family here. I don’t speak to them daily or weekly. Also, with friends, [ … ] I told them I quit, but many of them still smoke and they don’t think of stimulating, motivating or supporting me. It’s more like they think, well … [ … ] So, something is lacking from my direct environment. [R02]*

*I don’t have someone close to me that makes me think: “That one is going to pull me through it.” You understand? Intensive counselling or something. I really need someone. [R06]*

### Follow-up support

Respondents typically expressed the need for continued support after the campaign. This need manifested once they realized that the trick of Stoptober’s *SMART goal* was no longer effective, given their personal goal of quitting for good.

*I believe that those 28 days, those were the starting point, also for me. But well, you cannot expect it all to be gone afterwards. But, it is true that the physical part is over then, all discomforts, that is true. [R17]*

*But, afterwards it feels like going into the deep end. [ … ] Why don’t they continue? Why that month? That is just not smoking for a month and lighting your cigarette again afterwards. And hoping that people won’t do that. No, you have to push through then! [R08]*

These accounts indicate that the *SMART goal* had indeed contributed to the respondents’ self-efficacy, but it was not sufficient for most of them to continue as a non-smoker on their own.

Respondents suggested three types of continued smoking cessation support. First, the current Stoptober support could be prolonged, for example by continuing with the app, Facebook and related activities after the campaign.

*The Stoptober app should continue. I have another app like that and that one keeps counting how long you have been abstinent, how many cigarettes, how much money you saved. [ … ] I would certainly do that. [R08]*

Second, respondents illustrated how Stoptober participants could continue to support each other after the campaign through the online or offline social networks they had established during the campaign.

*Someone started a private [Facebook] group: We quit smoking 2016. 539 members. There are also posts. Well, then you read those messages. It just helps. It is really useful. [R03]*

Third, respondents thought Stoptober could connect to regular smoking-cessation services and stimulate participants to make use of available follow-up support, such as individual counseling or group support.

*And I think that if you, at that moment, use a powerful tool, like: “Listen everyone, well done, keep holding on”. That you give a good follow up. [ … ] That you get some more advice: “Here you can sign up for this training”. Very practical tools. [R20]*

Despite their critical remarks and suggestions for improvement, in general, respondents believed that in line with *social contagion theory*, Stoptober could play an important role in the normalization of smoking cessation.

*I think the initiative is good, because [smoking] is a problem and it is good that there is an official organization stepping forward that helps people [with smoking cessation], because if it is just constantly tolerated and accepted [ … ]. It is good to have this wake-up call. [ … ] [R20]*

## Discussion

### Main findings

Our retrospective interviews with Stoptober participants indicate that the temporary abstinence campaign largely functioned according to its theoretical principles. Program components built on social contagion theory familiarized respondents with Stoptober and motivated them to participate, the SMART principle points embedded in the program assisted them to set an achievable intermediate goal, and the variety of support offered in line with PRIME theory helped them to succeed with their temporary attempt to quit. However, once Stoptober was over, follow-up support was needed to overcome the enduring challenge of smoking cessation and becoming a permanent non-smoker.

### Limitations

The main limitation of this study is the selectiveness of the study sample. Most respondents managed to remain abstinent during Stoptober and only two relapsed after the campaign had ended. Although the high abstinence rates in our sample seem to contrast with real life smoking cessation attempts [15], they most likely reflect the high three-month quit rates in our quantitative evaluation of Stoptober [6]. The high number of successful quitters in our study sample could have caused our respondents’ accounts to be overly positive towards Stoptober and the support they had received. However, we observed that our respondents were willing to express criticism about the campaign and the available program support, regardless of their current smoking status. Nonetheless, using quota sampling to include additional respondents who had not remained abstinent during or shortly after Stoptober could have revealed other information on campaign limitations.

Second, in correspondence with the quantitative evaluation of Stoptober [6], most of our respondents were female (20 females out of 23 respondents). Male participants may have had other perspectives on Stoptober. Literature indicates that females are generally less successful in quitting smoking compared to males [16]. It is suggested that females might need additional behavioral counselling to address mood variability, environmental cues and social support [16]. This may have caused our female respondents to express a relatively high need for these types of support.

Third, respondents were primarily recruited via a call on the Stoptober Facebook page. This may have caused an overrepresentation of participants who used Facebook as their main source of support. Nonetheless, our sample also included a number of respondents who admitted not being frequent Facebook users and who preferred the Stoptober app or other measures as a way to receive support and remain abstinent.

### Interpretation of results

Our results indicate that the Stoptober campaign functioned largely according to its theoretical principles. In theory-based evaluations, illustrating an intervention’s working mechanisms may count as additional support for its effectiveness [10]. This type of evidence can be especially important for national programs, such as Stoptober, that cannot be properly evaluated in controlled study designs. Added to available evidence [5, 6], our results support the continuation and wider implementation of the Stoptober campaign.

Our results illustrate how Stoptober’s SMART principle supported smokers to stay abstinent for a defined period of time. This is in accordance with Bandura’s theory [18], which explains that behavior change can be facilitated through increased self-efficacy by setting achievable goals for a graded task over a graded time interval. Hence, Stoptober may be seen as a valuable first step in becoming a non-smoker, since accomplishing a temporary attempt to quit has been found to noticeably increase both self-efficacy [6] and the chance of quitting for good [5]. However, after the campaign, our respondents tended to regard Stoptober’s temporary quit goal as “fooling yourself”, since they were confronted once again with their (still difficult) personal goal of quitting for good. Since permanent abstinence is typically the reason smokers participate in the campaign [17], and longer term support is generally needed to achieve that goal [19], we present the respondent’s suggestions for how to strengthen the impact of Stoptober below.

A first suggestion builds on social network theory. So far, Stoptober successfully applied this principle to create a mass quit attempt [5] and create an online platform for social comparison and social support (this study). In line with our respondents, studies on online smoking cessation support suggest that face-to-face contacts may have added value for remaining abstinent [20, 21], especially for smokers who lack social support in their own social circle [22]. To establish such contacts, Stoptober could encourage participants to use their current social networks to stop smoking collectively (e.g. together with work colleagues) or facilitate participants building an additional social network (e.g. through local Stoptober meetings).

Two other suggestions are built on PRIME theory. In line with our respondents, studies on online or smartphone based smoking cessation interventions [22–26] suggest that improving the interactivity and tailoring of support could further increase Stoptober’s effectiveness. Our respondents also suggested that promoting local smoking cessation services could help Stoptober participants to overcome barriers to using these services [27] and increase the likelihood of taking advantage of professional support.

## Conclusions

Stoptober mainly functions according to its theoretical principles. Added to available evidence, this finding supports the continuation and wider implementation of the campaign. Connecting Stoptober to social networks and regular smoking-cessation services may improve the long-term abstinence rates of smokers after the campaign.

## Additional files

Additional file 1: Interview guide for participants in the Stoptober campaign. (DOC 37 kb)

Additional file 2: Illustration of data analysis process. Categorization of themes and codes into key psychological principles and into parts of the Results section. (DOCX 13 kb)

## Abbreviations

COREQ: Consolidated criteria for reporting qualitative research
PRIME: Plans, Responses, Impulses, Motives and Evaluations
SMART: Specific, Measurable, Attainable, Realistic and Time-sensitive

## References

[1] Tobacco: fact sheet [https://www.who.int/en/news-room/fact-sheets/detail/tobacco]. Accessed 3 Sept 2018.

[2] Prevalence of tobacco smoking [http://www.who.int/gho/tobacco/use/en/]. Accessed 3 Sept 2018.

[3] Bosdriesz JR, Willemsen MC, Stronks K, Kunst AE (2015). Socioeconomic inequalities in smoking cessation in 11 European countries from 1987 to 2012. J Epidemiol Community Health.

[4] Zhu S-H, Lee M, Zhuang Y-L, Gamst A, Wolfson T (2012). Interventions to increase smoking cessation at the population level: how much Progress has been made in the last two decades?. Tob Control.

[5] Brown J, Kotz D, Michie S, Stapleton J, Walmsley M, West R (2014). How effective and cost-effective was the national mass media smoking cessation campaign ‘Stoptober’?. Drug Alcohol Depend.

[6] Troelstra SA, Harting J, Kunst AE (2019). Effectiveness of a large, nation-wide smoking abstinence campaign in the Netherlands: a longitudinal study. Int J Environ Res Public Health.

[7] Christakis NA, Fowler JH (2007). The spread of obesity in a large social network over 32 years. N Engl J Med.

[8] Doran GT (1981). There's a S.M.a.R.T. way to write management's goals and objectives. Manag Rev.

[9] West Robert, Brown Jamie (2013). Theory of Addiction.

[10] Weiss CH. Evaluation: methods for studying programs and policies. 2nd ed. Upper Saddle River: Prentice Hall; 1998.

[11] Gray DE. Doing research in the real world. 3rd ed: London: SAGE Publications Ltd; 2014.

[12] Hennink MM, Kaiser BN, Marconi VC (2017). Code saturation versus meaning saturation:how many interviews are enough?. Qual Health Res.

[13] Galletta Anne, Cross William E. (2013). Conducting the Interview. Mastering the Semi-Structured Interview and Beyond.

[14] VERBI Software. MAXQDA. Berlin: VERBI Software; 2017.

[15] Hughes JR, Keely J, Naud S (2004). Shape of the relapse curve and long-term abstinence among untreated smokers. Addiction.

[16] Perkins KA (2001). Smoking cessation in women. Special considerations. CNS drugs.

[17] Troelstra SA, Harting J, Kunst AE (2017). Stoptober: Effecten op rookgedrag en ervaringen van deelnemers.

[18] Bandura A (1977). Self-efficacy: toward a unifying theory of behavioral change. Psychol Rev.

[19] Shahab L, McEwen A (2009). Online support for smoking cessation: a systematic review of the literature. Addiction.

[20] Cole-Lewis H, Perotte A, Galica K, Dreyer L, Griffith C, Schwarz M, Yun C, Patrick H, Coa K, Augustson E (2016). Social network behavior and engagement within a smoking cessation Facebook page. J Med Internet Res.

[21] Graham AL, Zhao K, Papandonatos GD, Erar B, Wang X, Amato MS, Cha S, Cohn AM, Pearson JL (2017). A prospective examination of online social network dynamics and smoking cessation. PLoS One.

[22] Bhattacharya A, Vilardaga R, Kientz JA, Munson SA (2017). Lessons from practice: designing tools to facilitate individualized support for quitting smoking. ACM Trans Comput Hum Interact.

[23] Fjeldsoe BS, Marshall AL, Miller YD (2009). Behavior change interventions delivered by Mobile telephone short-message service. Am J Prev Med.

[24] Lustria MLA, Noar SM, Cortese J, Van Stee SK, Glueckauf RL, Lee J (2013). A meta-analysis of web-delivered tailored health behavior change interventions. J Health Commun.

[25] Naslund JA, Kim SJ, Aschbrenner KA, McCulloch LJ, Brunette MF, Dallery J, Bartels SJ, Marsch LA (2017). Systematic review of social media interventions for smoking cessation. Addict Behav.

[26] Hébert ET, Stevens EM, Frank SG, Kendzor DE, Wetter DW, Zvolensky MJ, Buckner JD, Businelle MS (2018). An ecological momentary intervention for smoking cessation: the associations of just-in-time, tailored messages with lapse risk factors. Addict Behav.

[27] Vidrine Jennifer Irvin, Shete Sanjay, Cao Yumei, Greisinger Anthony, Harmonson Penny, Sharp Barry, Miles Lyndsay, Zbikowski Susan M., Wetter David W. (2013). Ask-Advise-Connect. JAMA Internal Medicine.

