# Supplementary material for: “Like you are fooling yourself”: how the “Stoptober” temporary abstinence campaign supports Dutch smokers attempting to quit
Source: BMC Public Health. 2019 May 7;19:522. doi: 10.1186/s12889-019-6833-y (PMC6505303; doi:10.1186/s12889-019-6833-y)
Supplement: Supplementary file 1 — Interview guide for participants in the Stoptober campaign. (DOC 37 kb) [file 12889_2019_6833_MOESM1_ESM.doc]

**Additional file 1. Interview guide for participants in the Stoptober campaign**

**Introduction**

Thank you for participating in our study! First, I will tell you more about myself and the study.

My name is [name] and I work as a researcher at the department of Public Health of the [research institute]. I am conducting research on the topic of smoking cessation. Right now, I am evaluating the Stoptober campaign.

Stoptober asked the [research institute] to evaluate the Stoptober campaign to assess its effects and to learn how the campaign could be improved. To understand how participants experience Stoptober and how the campaign supports smokers to quit, we are conducting interviews.

In the interview, we will talk about your quit attempt and your experiences with participating in Stoptober. We like to learn from this and to help other people who want to quit smoking. We will also talk about your opinion of Stoptober and ways to improve Stoptober.

As a researcher, I am independent from Stoptober. To me, it does not matter whether your quit attempt was successful or not or whether you are very negative of very positive about the campaign. I simply would like to hear how you feel about everything.

To make sure I will be able to use all things that we talked about during this interview I would like to make an audio recording. Are you okay with this?

[start audio recording]

Your participation in this study is anonymous. The audio recordings will be analyzed anonymously and we will not share your personal details with third parties.

If you want to withdraw your participation from this study, you can do this without telling us why.

Do you have any questions?

Do you consent to participate?

Okay, then we will start the interview.

**Interview**

First I would like to know how you are doing: Did you manage to stop smoking for 28 days?

Quitters

*Quitting process*

- How did you manage to quit smoking for 28 days?
- Are you still not smoking?

*Campaign experiences*

- To what extent did Stoptober help you to quit smoking?
- Which aspects of Stoptober made quitting easier to you? (app, facebook, twitter, vlogs, ambassadors, media attention)
- Which aspect was most helpful? In which way?

*Effect of campaign on social cognitive factors*

- How do you feel about smoking now? Did Stoptober change the way you feel about smoking?
- Are there people around you who also made a quit attempt recently?
- Do you think that your participation in Stoptober influenced the people around you?
- How do you experience situations where you used to smoke?
- Did you feel confident in the success of your quit attempt?
- During which moments did you experience difficulties with not smoking? (eating, drinking, friends, work, bars, restaurants, partner)
- What did you do when you craved a cigarette?
- How often do you think about smoking compared to the period before Stoptober?
- What do you do now during moments you used to smoke?
- Do you see yourself as a smoker? Why?
- To what extent did the people around you support you during your quit attempt?
- To what extent did you have contact with other Stoptober participants?

*Improving Stoptober*

- What would you tell someone who wants to quit smoking about Stoptober?
- How can Stoptober be improved?

*Future*

- How are you planning to continue in the next weeks and months?
- Is there anything you are afraid of?
- Is there anything you are confident in?

Sustained smokers

*Quitting process*

- Why do you think you did not manage to stop smoking?
- How long did you manage to remain abstinent?

*Campaign experiences*

- To what extent did Stoptober help you to quit smoking?
- Which aspects of Stoptober made quitting easier to you? (app, facebook, twitter, vlogs, ambassadors, media attention)
- Which aspect was most helpful? In which way?

*Effect of campaign on social cognitive factors*

- How do you feel about smoking now? Did Stoptober change the way you feel about smoking?
- Are there people around you who also made a quit attempt recently?
- Do you think that your participation in Stoptober influenced the people around you?
- Did you feel confident in the success of your quit attempt?
- During which moments did you experience difficulties with not smoking? (eating, drinking, friends, work, bars, restaurants, partner)
- What did you do when you craved a cigarette?
- How often do you think about smoking compared to the period before Stoptober?
- To want extent are you more conscious now of when and how often you smoke?
- Do you see yourself as a different type of smoker? Why?
- To what extent did the people around you support you during your quit attempt?
- To what extent did you have contact with other Stoptober participants?

*Improving Stoptober*

- What do you need to make a future quit attempt successful?
- What would you tell someone who wants to quit smoking about Stoptober?
- How can Stoptober be improved?

*Future*

- How are you planning to continue in the next weeks and months?
- Is there anything you are afraid of?
- Is there anything you are confident in?
