# Supplementary material for: “Like you are fooling yourself”: how the “Stoptober” temporary abstinence campaign supports Dutch smokers attempting to quit
Source: BMC Public Health. 2019 May 7;19:522. doi: 10.1186/s12889-019-6833-y (PMC6505303; doi:10.1186/s12889-019-6833-y)
Supplement: Supplementary file 2 — Illustration of data analysis process. Categorization of themes and codes into key psychological principles and into parts of the Results section. (DOCX 13 kb) [file 12889_2019_6833_MOESM2_ESM.docx]

**Additional file 2. Illustration of data analysis process**

Categorization of themes and codes into key psychological principles and into parts of the Results.

| **Parts of Results section**  (Subheadings in text) | **Key psychological principles**  (from Table 1) | **Themes in Coding scheme**  (from Table 3) |
| --- | --- | --- |
| **Support experienced** | Social contagion theory | *Prior to campaign*  Rationale to quit smoking  Rationale for participation in Stoptober  *Behavioral determinants*  Attitude  Social influence  Self-efficacy  Motivational strength  *Intervention components*  Mass media |
|  | SMART goals | *Prior to campaign*  Rationale to quit smoking  Rationale for participation in Stoptober  *Strategies to break habit*  Relapse prevention  Goal setting  *Behavioral determinants*  Social influence  Self-efficacy / Confidence in success  Motivational strength  *Intervention components*  Set date and time |
|  | PRIME theory | *Experiences during campaign*  Difficult moments  Positive experiences  *Strategies to break habit*  All codes  *Behavioral determinants*  Social influence  Self-efficacy  Habit  Identity  Motivational strength  *Intervention components*  Facebook  App  Ambassadors  Needs |
| **Further need for support** | Social contagion theory | *Behavioral determinants*  Social influence  *Intervention components*  Needs |
|  | SMART goals | *Strategies to break habit*  Relapse prevention  *Behavioral determinants*  Attitude  Self-efficacy  Motivational strength  *Intervention components*  Set date and time |
|  | PRIME theory | *Experiences during campaign*  Difficult moments  Additional support  *Strategies to break habit*  Relapse prevention  *Behavioral determinants*  Social influence  Self-efficacy  *Intervention components*  General  Facebook  App  Mass media  Ambassadors  Needs |
| **Follow-up support** | Social contagion theory | *Behavioral determinants*  Social influence  *Intervention components*  Needs |
|  | SMART goals | *Prior to campaign*  Rationale to quit smoking  Rationale for participation in Stoptober  *Strategies to break habit*  Goal setting  Relapse prevention  *Behavioral determinants*  Self-efficacy  *Intervention components*  Set date and time |
|  | PRIME theory | *Behavioral determinants*  Relapse prevention  *Future*  Confidence in maintaining abstinence High risk situations  *Intervention components*  Facebook  App  Needs |
